# Microbial Communities of Hydrothermal Guaymas Basin Surficial Sediment Profiled at 2 Millimeter-Scale Resolution

**DOI:** 10.3389/fmicb.2021.710881

**Published:** 2021-07-16

**Authors:** Bert Engelen, Tien Nguyen, Benedikt Heyerhoff, Saskia Kalenborn, Katharina Sydow, Houssem Tabai, Richard N. Peterson, Gunter Wegener, Andreas Teske

**Affiliations:** ^1^Institute for Chemistry and Biology of the Marine Environment, Carl von Ossietzky University of Oldenburg, Oldenburg, Germany; ^2^Department of Coastal and Marine Systems Science, Coastal Carolina University, Conway, SC, United States; ^3^MARUM – Center for Marine Environmental Sciences, University of Bremen, Bremen, Germany; ^4^Max Planck Institute for Marine Microbiology, Bremen, Germany; ^5^Department of Marine Sciences, University of North Carolina at Chapel Hill, Chapel Hill, NC, United States

**Keywords:** Guaymas basin, hydrothermal sediment, DGGE (Denaturating Gradient Gel Electrophoresis), 16S rRNA gene, mcrA (methyl-coenzyme M reductase A), amoA gene

## Abstract

The surficial hydrothermal sediments of Guaymas Basin harbor complex microbial communities where oxidative and reductive nitrogen, sulfur, and carbon-cycling populations and processes overlap and coexist. Here, we resolve microbial community profiles in hydrothermal sediment cores of Guaymas Basin on a scale of 2 millimeters, using Denaturing Gradient Gel Electrophoresis (DGGE) to visualize the rapid downcore changes among dominant bacteria and archaea. DGGE analysis of bacterial 16S rRNA gene amplicons identified free-living and syntrophic deltaproteobacterial sulfate-reducing bacteria, fermentative Cytophagales, members of the Chloroflexi (Thermoflexia), Aminicenantes, and uncultured sediment clades. The DGGE pattern indicates a gradually changing downcore community structure where small changes on a 2-millimeter scale accumulate to significantly changing populations within the top 4 cm sediment layer. Functional gene DGGE analyses identified anaerobic methane-oxidizing archaea (ANME) based on methyl-coenzyme M reductase genes, and members of the Betaproteobacteria and Thaumarchaeota based on bacterial and archaeal ammonia monooxygenase genes, respectively. The co-existence and overlapping habitat range of aerobic, nitrifying, sulfate-reducing and fermentative bacteria and archaea, including thermophiles, in the surficial sediments is consistent with dynamic redox and thermal gradients that sustain highly complex microbial communities in the hydrothermal sediments of Guaymas Basin.

## Introduction

Guaymas Basin, a young hydrothermally active spreading center in the central Gulf of California, differs from its open-ocean counterparts by its massive sediment layers that host deeply emplaced, hot volcanic sills. The resulting steep hydrothermal heat gradients are turning sedimentary organic matter under high temperature and pressure into hydrocarbons that enter hydrothermal circulation (Teske et al., 2014). The hydrocarbon-rich hydrothermal fluids reach the surficial sediments of Guaymas Basin, where they supply these subsurface-derived organic carbon sources and DIC to complex microbial communities that use seawater-derived electron acceptors, such as oxygen, nitrate and sulfate, to oxidize these carbon sources and to assimilate them into microbial biomass (Pearson et al., 2005). Metagenomic surveys of Guaymas Basin sediments recovered abundant hydrocarbon-utilizing and fermentative heterotrophic microbial communities (Dombrowski et al., 2017, 2018; Seitz et al., 2019). At the same time, inorganic hydrothermal energy sources such as sulfide, hydrogen, ammonia and methane (Von Damm et al., 1985) are used by chemosynthetic microbial communities – for example, sulfur-oxidizing filamentous mats or sulfur-oxidizing endobionts of marine invertebrates - that support the benthic ecosystem (Portail et al., 2015). The overlap of hydrothermal carbon sources and electron donors with seawater-derived electron acceptors and sedimenting organic matter sustains microbial interface communities where bacteria and archaea with divergent or mutually exclusive metabolisms coexist, for example nitrate-reducing sulfur-oxidizing filamentous bacteria of the family *Beggiatoaceae* and aerobic ammonia-oxidizing Thaumarchaeota (Winkel et al., 2014).

The question emerges whether the microbial communities of the uppermost sediment layers are completely mixed, as a consequence of hydrothermal circulation, bioturbation and microbial motility, or whether surficial hydrothermal sediments retain layered microbial communities that show some degree of depth stratification even on highly compressed spatial scales, in analogy to benthic microbial mats (Teske and Stahl, 2002). Published 16S rRNA gene-based or functional gene-based sequencing studies of Guaymas Basin sediments usually derived from sampling schemes where sediment cores are sliced into segments of one or several centimeters before DNA/RNA extraction and sequencing (Dhillon et al., 2003, 2005; Biddle et al., 2012; McKay et al., 2012, 2016; Vigneron et al., 2013; Cruaud et al., 2015; Dowell et al., 2016). These sampling schemes are frequently chosen based on the sample volumes required for parallel geochemical or process rate measurements. However, redox gradients in surficial sediments unfold on a scale of millimeters. So far, all published oxygen profiles from Guaymas Basin consistently indicate oxygen depletion within ca. 1–2 millimeters in hydrothermal sediments (Winkel et al., 2014), filamentous microbial mats (Schutte et al., 2018), or mat-covered hydrothermal sediments where small-scale “bubbles” or millimeter-sized local oxygen maxima within the upper centimeter indicate seawater inmixing (Gundersen et al., 1992; Teske et al., 2016). Centimeter-sized sampling schemes cannot track microbial population changes on this detailed scale, and is likely to yield predominantly anaerobic microbial populations even in surficial samples.

Here, we analyze bacterial and archaeal populations in Guaymas Basin sediments in approximately 2 mm downcore resolution, using Denaturing Gradient Gel Electrophoresis (DGGE) to separate PCR-amplified 16S rRNA and functional gene sequences of dominant phylotypes. Essentially a molecular fingerprinting method, DGGE separates PCR amplicons into different phylotypes with distinct migration patterns in denaturing gels that can be visually matched side by side, and probed or excised and sequenced for closer identification (Muyzer and Smalla, 1998; Strathdee and Free, 2013). The sequence yield and diversity spectrum of DGGE bands is not at all comparable with high-throughput sequencing surveys (for Guaymas Basin examples, see Biddle et al., 2012; Cruaud et al., 2015; Dowell et al., 2016; Teske et al., 2019, 2021; Ramírez et al., 2020), but still gives an overview on the most abundant community members. The visual comparison of community patterns allows targeted sequencing of DGGE bands that reflect the differences between the investigated samples. Thus, the prospect of visualizing microbial community changes in steep hydrothermal gradients could not be ignored, and motivated this case study.

## Materials and Methods

### Sampling

Two hydrothermal sediment cores (No. 4868-7 and 4868-10) were collected at a depth of 2003 m during *Alvin* dive 4868 on December 19, 2016, at the Mat Mound Massif in the southern trough of Guaymas Basin [27°00.40’N/111°24.57’W] (Pilot: Pat Hickey; Portside Observer, Richard Peterson; Starboard Observer: Leigha Peterson). In the sampling area, hydrothermal sediments were overgrown with microbial mats, and occurred interlaced with hydrothermal mineral precipitates that provided holdfasts for *Riftia* tube worms. Thermal gradient measurements with the *Alvin* heatflow probe showed that *in situ* temperatures at the sampling location, starting at bottom water temperature of 3°C right above the sediment surface, reached 60°C at 10 cm depth, 100°C at 20 cm depth, 112°C at 30 cm depth and 115°C at 40 and 50 cm depth (rounded values within 1°C). In core 4868-7, a total of 28 cm dark-brown sediment was recovered; gas voids appeared below 3.8 cm. The sediment changed from very soft, non-consolidated to fine-grained, more consolidated sediment at 12.7 cm, still containing gas voids. The overlying orange *Beggiatoaceae* mat was collected, and the sediment core (inner diameter 6.25 cm) was frozen at −80°C immediately after shipboard recovery, and transported frozen to the Institute for the Chemistry and Biology of the Marine Environment (ICBM) at Oldenburg University, Oldenburg, Germany.

The parallel core from the same site (4868-10) was used for rhizon sampling of porewater (Rhizosphere Research Products, Wageningen, Netherlands) as described previously (Seeberg-Elverfeldt et al., 2005). The overlying water was removed from the cores and holes were drilled into designated sediment sampling depths. Pretreated rhizons were injected and suction was applied with syringes for approx. 30 min. For sulfide analysis, 1 ml porewater subsamples were fixed with 0.1 ml of 0.1 M zinc acetate solution to preserve the sulfide as zinc sulfide until analysis by the methylene blue method (Cline, 1969). Porewater samples were also used for measuring stable ion concentrations of sulfate, nitrite, nitrate, phosphate, dissolved silica, and chloride using ion chromatography (Metrohm 930 Compact IC flex oven, Metrosep A PCC HC/4.0 preconcentration column, and Metrosep A Supp 5 Guard/4.0 chromatography column).

The microbiology core was gradually thawed at room temperature and sliced into approx. 2 mm intervals, by pushing the sediment upwards with a custom-designed piston. The highly fluid sediment was pushed to the rim of the core liner, and a 2 mm interval was siphoned off with 1 ml pipet tips [tips cut off for a wider opening] and collected for DNA extraction, before pushing the remaining sediment core again to the rim of the core liner and sampling the next layer; sediment granules and inhomogeneities prohibited sampling on a finer scale. The procedure was repeated until the upper 4 cm of the core were collected in approx. 2 millimeter intervals; deeper layers were collected in 4 millimeter intervals. The highly fluid sediment surface was dominated by supernatant water and possibly porewater that had frozen out of the core and diluted the sediment; this mixed surface layer was siphoned off while the piston was gently pushed up in two intervals (0–0.14 cm, 0.14–0.29 cm). The sediment in the proper sense started at 3 mm depth; this cutoff value was chosen for the DGGE analyses. The sediment contained small clumps and carbonate concretions that interfered with exact 2 mm slicing; therefore the depth of each sediment sample layer was checked and recalculated based on sample volume: 0.29 – 0.48 cm, 0.48 – 0.66 cm, 0.66 – 0.82 cm, 0.82 – 1.02 cm, 1.02 – 1.21 cm, 1.21 – 1.43 cm, 1.43 – 1.62 cm, 1.62 – 1.85 cm, 1.85 – 2.08 cm, 2.08 – 2.28 cm, 2.28 – 2.44 cm, 2.44 – 2.64 cm, 2.64 – 2.85 cm, 2.85 – 3.04 cm, 3.04 – 3.25 cm, 3.25 – 3.45 cm, 3.45 – 3.63 cm, 3.63 – 3.83 cm, 3.83 – 4.02 cm, 4.02 – 4.20 cm. The depth values are rounded to full or half millimeters in the DGGE figures. The upper two sediment layers contained some orange-colored *Beggiatoaceae* filaments that were left behind after lifting off the mat and freezing the core.

### DNA Extraction and PCR

DNA was extracted from each sediment sample using the DNeasy power soil kit (Qiagen, United States), following the manufacturer’s instructions, except 0.5 g instead of 0.25 g of sample were used. DNA extracts were quantified using NanoDrop (Supplementary Figure 1). For PCR, 1 μl of 1:10 diluted DNA was used, to dilute inhibitors. Primer sets for PCR were selected to amplify gene segments of approx. 500 basepairs, to combine sufficient sequence information for phylogenetic resolution with adequate stability in the DGGE gel denaturing gradient.

A 550 bp segment of bacterial 16S rRNA genes was amplified using the forward primer GC-357f extended with the underlined GC-clamp (5′-CGC CCG CCG CGC CCC GCG CCC GGC CCG CCG CCC CCG CCC CCC TAC GGG AGG CAG CAG-3′), and the reverse primer 907R (5′-CCG TCA ATT CCT TTG AGT TT-3′), slightly modified versions of a previously published bacterial 16S rRNA gene primer pair (Muyzer et al., 1995). A 499 bp segment of the key gene of methanogenesis and methane oxidation, methyl coenzyme M transferase Alpha subunit (mcrA), was amplified with primer combination mcrA1f (5′-TMG GAT TCA CAC ART AYG CWA CAG C-3′) as the forward primer, and mcrA500R –GC (5′-CGC CCG CCG CGC CCC GCG CCC GTC CCG CCG CCC C CG CCT TCA TTG CRT AGT TWG GRT AGT T-3′) as a reverse primer with the GC-clamp (Luton et al., 2002; Wilms et al., 2007). Ammonia monooxygenase sequences were amplified using primers Arch-amoAF (5′-STA ATG GTC TGG CTT AGA CG-3′) and Arch-amoAR (5′-GCG GCC ATC CAT CTG TAT GT-3′) (Francis et al., 2005) to obtain 635 bp segments of archaeal amoA genes, and primers amoA-322F (5′-CCC CTC KGS AAA GCC TTC TTC-3′) and amoA-822R (5′-CCC CTC KGS AAA GCC TTC TTC-3′) to obtain 453 bp segments of betaproteobacterial amoA genes (Rotthauwe et al., 1997; Junier et al., 2010). The PCR mastermix (50 μl) contained 38.6 μl PCR-water, 5 μl Taq buffer (10×), 0.4 μl dNTP-Mix (25 mM, each), 1 μl MgCl_2_ (25 mM), 1 μl BSA (10 mg/ml), 1 μl MolTaq (1 U/μl), 2 × 1 μl of the respective primers (10 pmol/μl) and 1 μl of DNA template.

Thermal cycling was performed with MasterCycler^®^ Gradient Thermal Cycler (Eppendorf AG, Hamburg, Germany), with the following parameters for amplification of 16S rRNA gene fragments: 96°C initial hold for 4 min to activate the Taq polymerase (MolTaq, Molzym, Germany), followed by 37 cycles of amplification, with each cycle consisting of 30 s of denaturation at 96°C, 45 s of annealing at 57°C, and 1 min extension at 72°C, and concluded with a final extension step of 10 min at 72°C. Amplification conditions for mcrA genes were identical, except the annealing temperature was adjusted to 56°C, and the amplification was performed by 30 cycles. PCR conditions for archaeal amoA gene amplification were adjusted to an initial hold at 94°C for 5 min, and 46 cycles consisting of denaturation at 94°C for 45 s, annealing at 53°C for 1 min, and extension at 72°C for 1 min (modified after Francis et al., 2005). PCR conditions for bacterial amoA gene amplification were adjusted to an initial hold at 94°C for 3 min, and 42 cycles consisting of denaturation at 94°C for 45 s, annealing at 57°C for 1 min, and extension at 72°C for 1 min (modified after Rotthauwe et al., 1997). PCR products were checked with gel electrophoresis using a 1.5% agarose gel to ensure that each primer combination generated a single band of appropriate length.

### Denaturing Gradient Gel Electrophoresis

The DGGE analysis was performed on an INGENYphorU-2 system (Ingeny, Goes, Netherlands). In the DGGE gel, 30 μl of each PCR sample plus 5 μl of buffer were loaded into the central gel slots, and 15 μl of marker and 5 μl of loading buffer were loaded on both sides of the gel. DGGE was performed with PCR products loaded onto 6% polyacrylamide gels in 1× TAE buffer (40 mM Tris, 1 mM EDTA, pH 7.4) with a denaturing gradient from 40 to 70% (with 100% denaturant corresponding to 7 M urea and 40% formamide) at 100 V and 60°C for 20 h for bacterial 16S rRNA genes and mcrA genes. The denaturant gradient and running time were adjusted to 15–55% and 15 h for archaeal amoA genes, and to 30–60% and 20 h for bacterial amoA genes. After the gradient gel had been poured with a gradient mixer, it was topped with a denaturant-free gel portion that surrounded the teeth of the DGGE comb (forming the PCR product loading slots) to facilitate the start of migration of the PCR products through the DGGE gel. We note that DGGE analyses of archaeal 16S rRNA gene fragments were attempted repeatedly, but did not result in clearly discernible DGGE patterns or defined DGGE bands, and are therefore not included in this study.

After the DGGE runs, the gel bands were stained using SybrGold (Molecular probes, Eugene, OR, United States) for 1–2 h after completion of electrophoresis. The band patterns were analyzed by cluster analysis using the software package GelComparII (Applied Maths NV, 1992, Sint-Martens-Latem, Belgium) on DGGE gel images taken under UV-light as described (Wilms et al., 2006). Small gel pieces containing bands of specific PCR products were excised. The gel pieces were incubated overnight in 50 μl PCR water at 4°C. After DGGE separation, PCR products were reamplified using the same primers and PCR conditions as before, except for a decrease of the annealing temperatures by 2°C for all primer combinations and without the GC-clampx attached to the forward primer. Subsequently, the amplicons were purified using the QIAquick^®^ PCR Purification kit (Qiagen, N.V., Hilden, Germany). Then, 5 μl of PCR product were mixed with 5 μl of forward primer (5 pmol/μl), and submitted to GATC Biotech AG, Konstanz, Germany, for sequencing.

### Sequence Analysis

Each DGGE band sequence was checked against related sequences of cultured and uncultured bacteria and archaea in GenBank using BLASTN (Altschul et al., 1990). Phylogenetic trees for DGGE band sequences and for related sequences of well-documented and published phylotypes and cultures from GenBank were inferred with the program package PAUP4.0 (Swofford, 2000), using HKW85 distances, transition and transversion rates assumed to follow gamma distribution with shape parameter = 0.5, and Minimum Evolution as optimality criterion. Branching patterns were checked with 1000 bootstrap reruns.

## Results

### Thermal and Geochemical Characterization

**FIGURE 1:**
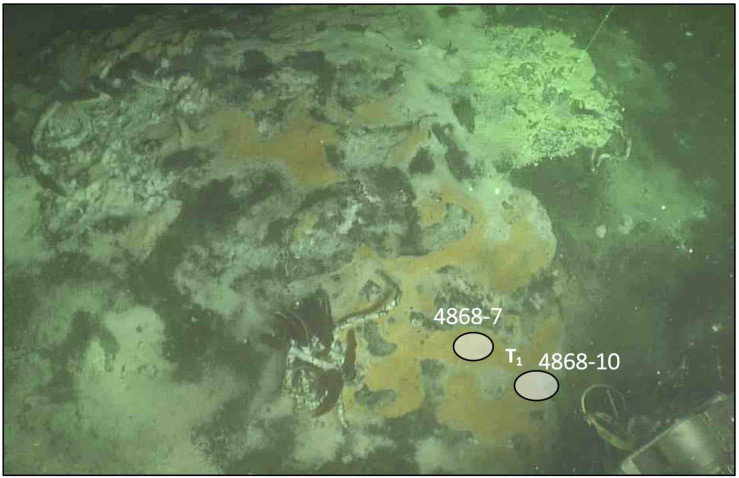
Hydrothermal sediments with white, yellow and orange sulfur-oxidizing microbial mats (*Beggiatoaceae*), sulfur precipitates, and *Riftia* tubeworm colonies at Mat Mound Massif (111°24.57’W/27°00.40’N). The DGGE core 4868-7 and the porewater geochemistry core 4868-10 were collected next to the temperature profile measured using the *Alvin* heatflow probe (Supplementary Table 1). The two green laser dots in the center of the image above the coring sites mark a distance of 10 cm. Image obtained from *Alvin* framegrabber, WHOI.

Thermal gradients show that the sampling site is hydrothermally active, consistent with conspicuous mat sulfur-oxidizing white, yellow and orange *Beggiatoaceae* mats (Figure 1) that indicate hydrothermal activity (McKay et al., 2012). The temperature reached 100°C within 20 cm, and rises to 115°C within the 50 cm-length of the heat flow probe. Adjacent to hydrothermal sediment core 4868-7 that was used for DGGE analysis, core 4868-10 was sampled for parallel geochemical characterization of the sampling site by porewater analysis (Table 1). The porewater concentrations are consistent with hydrothermal activity. Ammonia concentrations in the millimolar range, and nitrite and nitrate concentrations in the lower micromolar range are similar to other hydrothermal sediment cores of Guaymas Basin where ammonia-rich hydrothermal fluids are mixing with traces of nitrite and nitrate that most likely originate in seawater or in nitrate-accumulating *Beggiatoaceae* mats overlying the sediment surface (see data compilations in Buckley et al., 2019, and in Schutte et al., 2018, Supplementary Materials). Phosphate concentrations are generally low in Guaymas Basin hydrothermal cores; the 1–5 micromolar concentrations observed here are comparable with those in other Guaymas Basin surveys (Teske et al., 2021). Silica concentrations reached ca. 0.26 mM at the surface, where they exceeded deep water column concentrations of 0.175 mM (Campbell and Gieskes, 1984), and increased toward 0.5 mM below the sediment surface (Table 1). Increasing silica concentrations are indicative of hydrothermal silica dissolution and mobilization from diatom-rich sediments (Calvert, 1966). Hydrothermal silica dissolution occurs within a temperature window of 100–150°C, consistent with the local thermal regime (Peter and Scott, 1988). Sulfate remains available in >10 millimolar concentrations throughout the core, consistent with previous analyses that indicate extensive sulfate penetration of hydrothermal cores, presumably due to hydrothermal circulation and seawater inmixing (McKay et al., 2012). The sediments also contained approx. 3 mM sulfide, typical for sulfidic sediments with sulfur-oxidizing *Beggiatoaceae* mats (McKay et al., 2012).

**TABLE 1:** Porewater concentrations for core 4868-10.

samples	NH_4_^+^ [μM]	NO_2_^–^ [μM]	NO_3_^–^ [μM]	PO_4_^3–^ [μM]	SiO_4_^4–^ [μM]	SO_4_^2–^ [mM]	HS^–^/S^2–^ [mM]	Cl^–^ [mM]
SN*	247	0.583	9.7	1.735	103	30.4	0.961	556.3
1 cm	1633	0.861	10.072	1.076	267	21.6	3.201	564.0
3 cm	1223	0.757	6.38	0.903	261	22.5	2.833	536.8
5 cm	4055	0.501	5.967	1.224	385	11.4	no data	577.9
7 cm	1220	0.58	7.745	5.744	451	24.2	no data	567.0

The sample names represent the injection depths of rhizon samplers.*SN = supernatant.

### DNA Yield for DGGE

Sufficient amounts of DNA for PCR amplification and DGGE analysis of 16S rRNA genes and functional genes was obtained from the surficial sediment down to 2 to 4 cm depth, depending on the specific target gene. Concentrations of extracted DNA within the upper 2 cm sediment layers exceeded those in deeper sediment layers by one order of magnitude (Supplementary Figure 1).

### Bacterial 16S rRNA Genes

**FIGURE 2:**
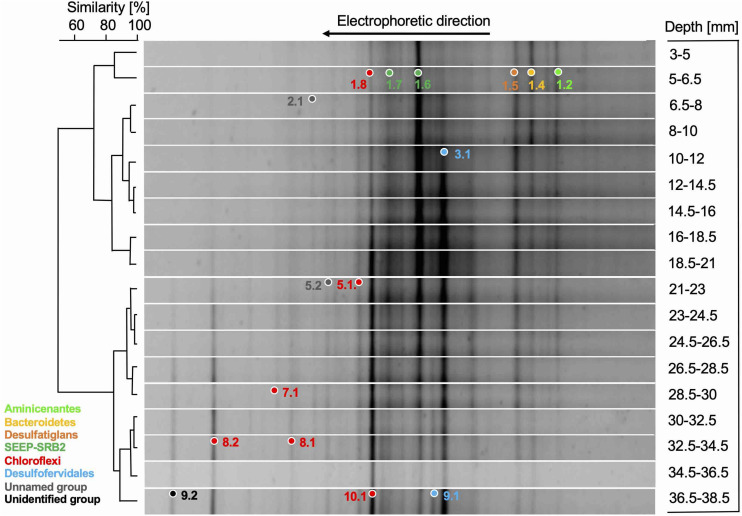
DGGE analysis of bacterial 16S rRNA genes from sediment core 4868-7, with similarity clustering pattern and positions of excised and sequenced DGGE bands, color-coded to match their phylogeny. The DGGE lanes of the deepest PCR-positive sediment samples, 38–40 and 40–42 mm, showed the same pattern as the 36–38 mm layer, but the bands were faint and the DGGE lanes are not shown.

**FIGURE 3:**
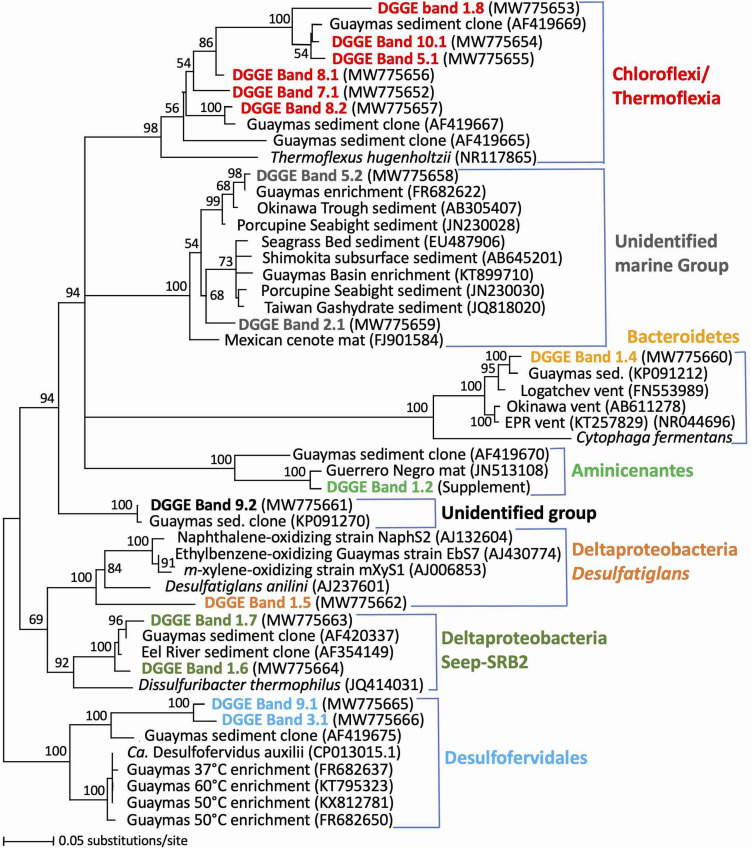
Distance phylogeny of bacterial 16S rRNA gene DGGE fragments based on complete amplicon length minus primer sequences, color-coded to match the DGGE bands in Figure 2. The sequence of DGGE band 1.2 is provided in Supplementary Table 2. Bootstrap support for nodes (in%) was derived from 1000 iterations. The scale bar in this and subsequent phylogenies shows sequence distance (Minimum Evolution) as specified in Materials and Methods.

Denaturing Gradient Gel Electrophoresis analysis of bacterial 16S rRNA genes showed a gradual transition of different dominant bands appearing and disappearing within the upper 4 cm; the DGGE pattern was further resolved by cluster analyses into three distinct clusters that ranged from 2 to 7 mm, 7 to 21, and below 21 millimeters depth, respectively (Figure 2). This clustering pattern suggests that the microbial community is shaped by thermal or geochemical factors, for example the presence of oxygen and nitrate that penetrate a few millimeters into the sediment (Winkel et al., 2014; Teske et al., 2016). Within the top sediment layers, DGGE bands represented distinct phylogenetic and physiological groups (Figure 3), such as heterotrophic, possibly fermentative Aminicenantes (Kadnikov et al., 2019) and Cytophagales (Garcia-López et al., 2019), sulfate-reducing Deltaproteobacteria of the aromatics-oxidizing *Desulfatiglans* lineage (reviewed in Teske, 2019) and the alkane-oxidizing SEEP-SRB2 lineage (Krukenberg et al., 2018). While they decreased gradually below 2 cm depth, DGGE bands remained visible in deeper sediment layers as well (Figure 2). Within the upper cm, the thermophilic Desulfofervidales lineage (formerly Hotseep-1) appeared gradually in the DGGE pattern and persisted undiminished throughout the deeper sediments; this lineage is currently represented by the facultatively syntrophic, H_2_-oxidizing sulfate-reducing bacterium *Candidatus* Desulfofervidus auxilii that thrives at temperatures of 50 to 60°C (Krukenberg et al., 2016). The occurrence pattern of this band in the hydrothermal sediments is consistent with thermophilic habitat preference. Several DGGE bands represented the class *Thermoflexia* within the phylum *Chloroflexi*, named for the heterotrophic, microaerophilic and facultatively anaerobic thermophile *Thermoflexus hugenholtzii* from terrestrial hot springs (Dodsworth et al., 2014). Some *Thermoflexia* phylotypes persisted throughout all analyzed sediment layers [bands 1.8/10.1], others appeared prominently below 2 cm depth [band 8.2]. Several bacterial lineages could not be assigned to previously described lineages, including a phylotype [band 9.2] that is appearing below 2 cm depth, and members of an uncultured subsurface lineage [bands 2.1 and 5.2] that appeared as faint bands throughout the DGGE pattern (Figure 2). These phylogenetically divergent DGGE bands are closely matching PCR-amplified 16S rRNA gene sequences from Guaymas Basin sediments (Teske et al., 2002; Dowell et al., 2016) and hydrocarbon-oxidizing enrichments (reviewed in Teske, 2019), indicating that these phylotypes represent undescribed bacterial lineages that are recovered consistently from Guaymas Basin sediments during different sampling and sequencing surveys (Figure 3).

### Ammonia-Oxidizing Bacteria and Archaea

**FIGURE 4:**
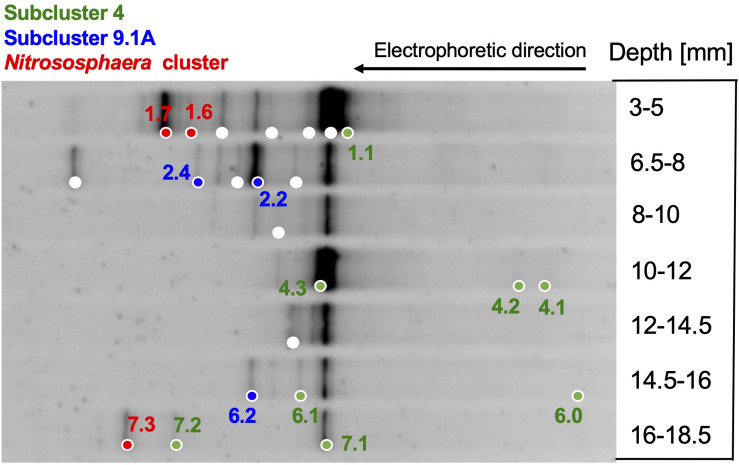
DGGE analysis of archaeal ammonia monooxygenase alpha subunit genes (amoA). DGGE bands are color-coded to match their phylogeny. Red, *Nitrososphaera* cluster; green, subcluster 4; blue, subcluster 9.1A (based on Pester et al., 2012). No amplifiable archaeal amoA genes were found below ca. 2 cm depth.

**FIGURE 5:**
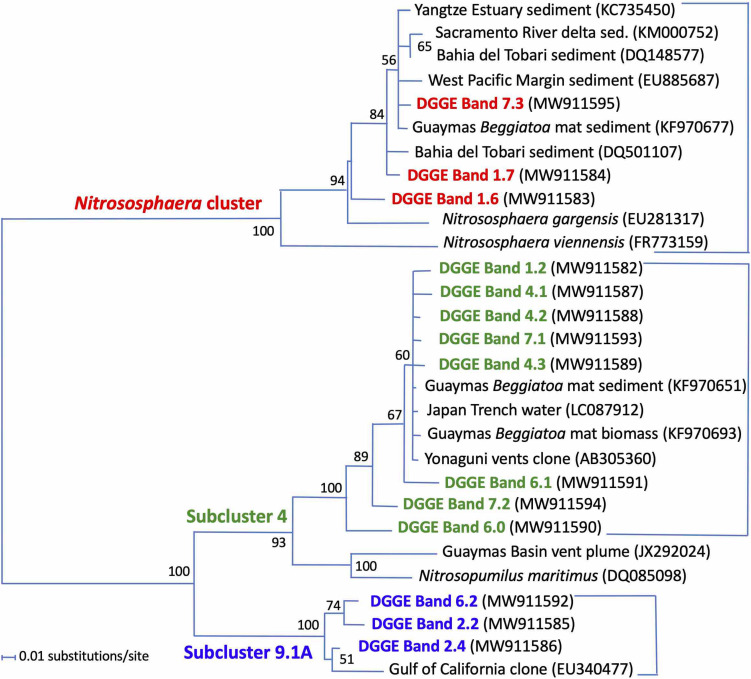
Distance phylogeny of archaeal amoA gene DGGE fragments, based on complete amplicon length minus primer sequences, color-coded to match the DGGE bands in Figure 4. Bootstrap support for nodes (in%) was derived from 1000 iterations.

Ammonia monooxygenase genes are functionally essential for bacteria and archaea that oxidize ammonia to nitrite, and form different phylogenetic lineages – requiring different PCR primers – among the bacteria and the archaea (Purkhold et al., 2000; Junier et al., 2010; Alves et al., 2018). Detecting amoA phylotypes is consistent with the abundance of ammonia in hydrothermal fluids (Table 1), and with ammonia produced via nitrate and nitrite reduction by *Beggiatoaceae* mats (Schutte et al., 2018). Archaeal amoA genes are detectable until ca. 18 mm depth (Figure 4) and belong to three different lineages (Figure 5): a cluster related to cultured ammonia-oxidizing archaea of the genus *Nitrososphaera*, especially the moderately thermophilic hot spring species *Nitrososphaera gargensis* (Hatzenpichler et al., 2008); and the environmentally widespread, uncultured subclusters 4 and 9.1, as defined by large-scale gene and clustering surveys from terrestrial soils (Pester et al., 2012). The Guaymas Basin sediment phylotypes are not specifically affiliated with the pelagic ammonia oxidizer *Nitrosopumilus maritimus* (Könneke et al., 2005; Qin et al., 2017), but they are closely related to archaeal amoA phylotypes that were previously recovered from sulfur-oxidizing orange *Beggiatoaceae* mats in Guaymas Basin, and from sediments underlying these mats (Winkel et al., 2014). The three clusters of DGGE phylotypes do not show a specific depth distribution.

**FIGURE 6:**
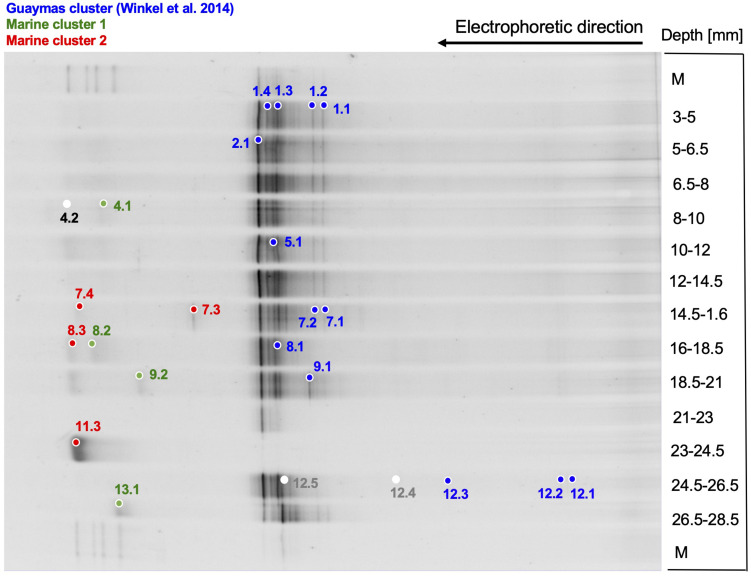
DGGE analysis of bacterial ammonia monooxygenase alpha subunit genes (amoA). DGGE bands are color-coded to match their phylogeny. The Guaymas cluster in blue was defined by Winkel et al. (2014). Marine clusters 1 and 2, highlighted in green and red, are defined in this publication.

**FIGURE 7:**
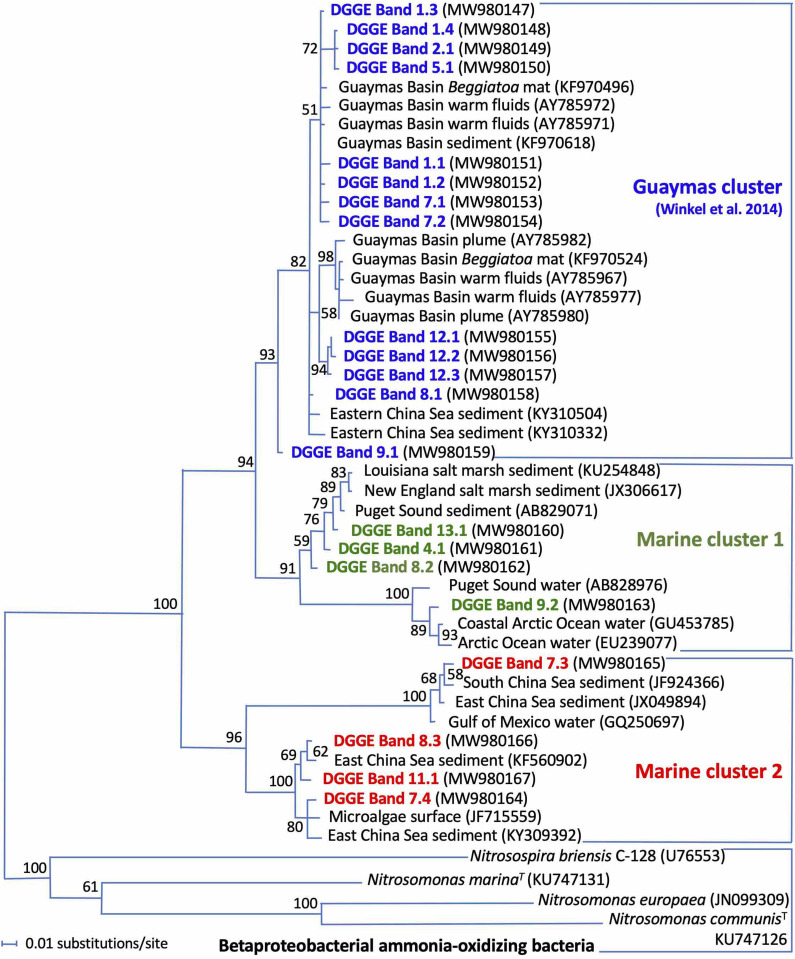
Distance phylogeny of bacterial amoA gene DGGE fragments, based on complete amplicon length minus primer sequences, color-coded to match the DGGE bands in Figure 6. Bootstrap support for nodes (in%) was derived from 1000 iterations.

Bacterial amoA genes were detectable within the upper 28 mm of the sediment, and thus extended ca. 1 cm deeper into the sediment than their archaeal counterparts (Figure 6). The closest cultured counterparts of the Guaymas DGGE sequences are amoA genes of marine betaproteobacterial ammonia-oxidizing bacteria of the genera *Nitrosospira* and *Nitrosomonas* (Figure 7). However, the DGGE phylotypes fall into at least three separate branches without cultured representatives (Figure 7): The Guaymas cluster of marine amoA genes harboring previously obtained phylotypes from Guaymas Basin hydrothermal sediments, *Beggiatoaceae* mats, and warm vent fluids (Winkel et al., 2014) was found in all DGGE samples except one (Figure 6). Two uncultured groups, termed Marine clusters 1 and 2, contained amoA phylotypes from globally diverse marine water and sediment samples (Figure 7); these amoA types were represented by mostly faint DGGE bands in sediment layers below 8 mm depth (Figure 6).

### Methane-Oxidizing Archaea

**FIGURE 8:**
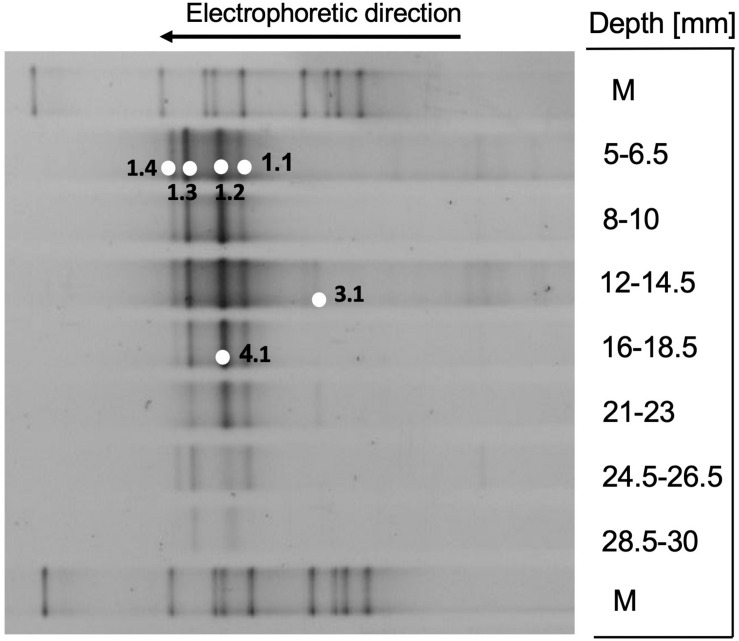
DGGE analysis of methyl-coenzyme M reductase alpha subunit genes (mcrA).

**FIGURE 9:**
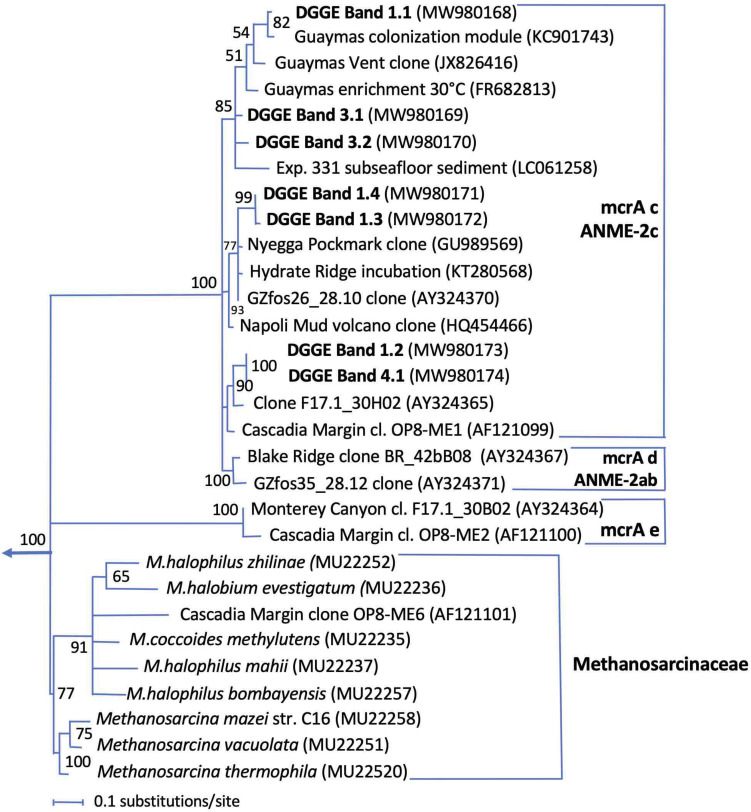
Distance phylogeny of mcrA gene DGGE fragments, based on complete amplicon length minus primer sequences, color-coded to match the DGGE bands in Figure 8. Bootstrap support for nodes (in%) was derived from 1000 iterations.

The methyl-coenzyme M reductase alpha subunit gene, a key gene for methanogenesis and methane oxidation (Knittel and Boetius, 2009), was detected by DGGE between 0.5 and 3 cm depth, without major changes in downcore DGGE patterns (Figure 8). All mcrA DGGE bands were members of the mcrA gene cluster c (Figure 9), the equivalent to ANME-2c archaea in 16S rRNA gene-based phylogenies (Hallam et al., 2003; Trembath-Reichert et al., 2013). In previous mcrA gene surveys of Guaymas Basin sediments, ANME-2 archaea and their mcrA genes were preferentially detected in surficial, temperate sediments that are exposed to seawater inmixing (Biddle et al., 2012; McKay et al., 2016). Related mcrA genes were also obtained in methane-oxidizing enrichment cultures based on Guaymas sediments (Holler et al., 2011), and on *in situ* enrichments in microbial mast of Guaymas Basin (Callac et al., 2013).

We note that the mcrA gene primer combination that was used here does not include the ANME-1 lineage of anaerobic methane-oxidizing archaea which require a separate primer pair that remains to be tested for DGGE usage (Lever and Teske, 2015). The ANME-1 archaea are perhaps the most ubiquitous microbial community components of Guaymas Basin sediments and have appeared prominently in practically every single gene-based survey of Guaymas Basin sediments (see data compilation in Dowell et al., 2016). Their tolerance for sulfate-limited and geothermally heated sediment layers has been documented repeatedly (Vigneron et al., 2013; McKay et al., 2016).

## Discussion

### Surficial Sediment Characteristics

In this case study, the results of DNA extraction, PCR amplification and DGGE demonstrated that DNA of sufficient quantity or quality for this type of analysis was limited to the surficial centimeters of this Guaymas Basin core, approx. 2–4 cm depth depending on the gene target. The microbial populations that are profiled by DGGE are sandwiched into this narrow surface layer, although the thermal regime at the time of measurement would have permitted thermophilic or hyperthermophilic microbial life until at least 20 cm depth with *in situ* temperatures of 100°C (Supplementary Table 1). Even though this sampling site was selected largely for effective recovery of hydrothermal sediment (always a consideration during time-limited *Alvin* dives), without claims of having chosen a particularly representative site, its characteristics are generally consistent with previous surveys of Guaymas Basin hydrothermal sediments, where microbial abundance and activity are strongly concentrated toward the top few centimeters. Surficial sediments immediately below the seawater-sediment interface show the highest degree of microbial activity, as shown by peak rates of sulfate reduction (Elsgaard et al., 1994; Weber and Jørgensen, 2002; Meyer et al., 2013) and acetate oxidation (Zhuang et al., 2019), by rapid consumption of oxygen and nitrate (Gundersen et al., 1992; Winkel et al., 2014; Teske et al., 2016), and by microbial population peaks, reflected in cell count maxima (Meyer et al., 2013), concentration maxima of microbial lipids (Guezennec et al., 1996; Teske et al., 2002; Schouten et al., 2003), and dilution series of cultivable thermophiles (Teske et al., 2009). In this core, relative DNA yield is highest in the upper 2 cm, and then decreases by an order of magnitude (Supplementary Figure 1). To summarize, most microbial life in Guaymas Basin hydrothermal sediments is crowded into a few centimeters of surficial sediments, and the sediment core studied here by DGGE analysis underscores these results obtained by mutually independent investigations.

The thermal regime at the time of measurement would have permitted thermophilic or hyperthermophilic microbial life until at least 20 cm depth with *in situ* temperatures of 100°C; interpolating temperatures for 4 cm depth, the depth extent of DGGE analysis, would yield ca. 25–30°C (Supplementary Table 1). Even considering the possibility of incomplete DNA recovery or PCR inhibition, the measured temperature profile should have permitted that microbial populations and their representation in DGGE profiles extend more deeply into the sediment. However, time point measurements of steep thermal and geochemical gradients are not reporting fluctuating hydrothermal activity over several hours or days (McKay et al., 2012, 2016) or thermal flushing of organic matter and biomass from deeper sediments (Lin et al., 2017). Close examination of microbial mats on Guaymas Basin hydrothermal sediments often reveal crater-like structures and highly disturbed sediment with sulfur crusts in the center of hydrothermal areas (Teske et al., 2016) suggestive of short-term hydrothermal flushing or purges. Pulsating hydrothermal activity would effectively concentrate microbial cells and their activities within the upper few centimeter layers of active hydrothermal sediments.

### Microbial Coexistence in Surficial Sediments

**FIGURE 10:**
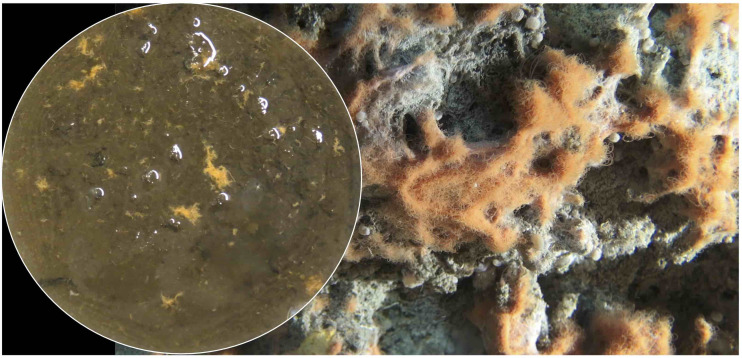
Close-up video still of Guaymas Basin sediment surface with orange *Beggiatoaceae* mats, obtained with the down-facing *Alvin* camera during dive 4872 (December 24, 2016). The contrasting insert shows the surface of sediment core 4868-7 in the lab after gradual thawing, and pipetting off supernatant. Insert and video still image were adjusted to approximately the same scale based on individual orange *Beggiatoaceae* filaments with diameters of ca. 30–40 micrometers.

The DGGE analysis shows the coexistence of microbial populations with aerobic and anaerobic metabolisms in the surficial sediments: aerobic ammonia-oxidizing Betaproteobacteria and marine Thaumarchaeota (Teske et al., 1994; Qin et al., 2017); sulfate-dependent methane-oxidizing ANME-2c archaea (Hallam et al., 2003); sulfate-reducing bacteria of the *Desulfatiglans* lineage that can perform complete oxidation of aromatic hydrocarbons (Teske, 2019); thermophilic hydrogen oxidizers of the Desulfofervidales (Krukenberg et al., 2016); syntrophic methane and short-chain alkane-oxidizing Desulfofervidales and SEEP-SRB2 (Krukenberg et al., 2018); microaerobic or fermentative, complex carbohydrate-degrading Cytophagales (Garcia-López et al., 2019), and microaerobic or fermentative, presumably thermophilic protein-degrading Thermoflexia (Dodsworth et al., 2014). Orange *Beggiatoaceae* in the upper sediment layers (Figure 10) perform sulfide oxidation coupled to nitrate/nitrite reduction to ammonia (Schutte et al., 2018), potentially alternating with the reduction of redox-intermediate sulfur compounds, such as tetrathionate (Buckley et al., 2019). The depth extent of coexisting aerobic and anaerobic populations was estimated based on DGGE patterns of functional genes of aerobic archaeal and bacterial ammonia oxidation that extend to 18 and to 28 millimeters depth, respectively. These aerobic populations would depend on inmixing of oxygenated seawater bubbles that have been repeatedly detected at approx. 1 cm depth in sediments underneath *Beggiatoaceae* mats in Guaymas Basin (Gundersen et al., 1992; Teske et al., 2016). DGGE analysis of 16S rRNA genes indicates that anaerobic and thermophilic bacterial populations (Thermoflexia, Desulfofervidales) extend deeper into the sediment, at least 4 cm.

### Methodological Limitations

In contrast to high-throughput sequencing approaches that yield 10^4^ to 10^5^ sequences per sample (see Teske et al., 2019 and Ramírez et al., 2020 for recent Guaymas Basin examples), the spatial resolution of DGGE gels limits amplicon separation and identification to approximately a few dozen phylotypes, which are visually tracked in different sampling layers and sediment depths. In addition to the difference in sequence yield and diversity, we note that microbial depth resolution in 2-millimeter depth intervals comes with its own inherent limitations. Sediments and microbial mats in the Guaymas Basin seafloor show fine-scale topography, small fluid channels and orifice-like openings that collapse during core retrieval, freezing and slicing. Most likely, a complex three-dimensional topography of microbial niche habitats and their inhabitants is reduced to an “averaged” layering of homogenized sediments, as shown by a visual comparison of the core surface before sampling in the lab, and sediments and mats photographed *in situ* by Alvin’s bottom-facing camera (Figure 10). This issue has obvious ramifications for disturbing the distribution of microbial cells and DNA in the sediment. Maintaining a similar interface landscape in the lab (using sulfur-oxidizing microbial mats from an easily sampled coastal model system) and mapping it with oxygen microelectrodes has revealed the importance of small-scale topography and surface structure (Møller et al., 1985). Possible solutions toward preserving the original microbial community architecture should include the fixation and FISH analysis of entire sediment slices containing multiple microbial consortia, as demonstrated in Sonora Margin cold seep sediments (Vigneron et al., 2014).

### Outlook

The spatial overlap of microorganisms with aerobic ammonia-oxidizing and anaerobic sulfate-reducing and methane-oxidizing microbial communities indicates fluctuating redox conditions in mat-covered surficial sediments, and is consistent with previous observations of a dynamic temperature regime in Guaymas Basin hydrothermal sediments (McKay et al., 2016); it is also consistent with predictions of this environmental niche based on the luxuriant growth of thick *Beggiatoaceae* mats that require simultaneous availability of electron acceptors and donors by turbulent mixing of seawater and vent fluids (Jannasch et al., 1989). Overlapping and/or fluctuating redox and thermal regimes in surficial sediments, and the simultaneous availability of different energy and carbon sources in highly compressed geochemical gradients could be a major reason for the high microbial diversity that characterizes Guaymas Basin. Already the first bacterial, archaeal and eukaryotic small subunit rRNA gene sequencing surveys had commented on the apparent overlap of microbial communities from hydrothermal vents, cold hydrocarbon seeps and organic-rich marine sediments in Guaymas Basin (Edgcomb et al., 2002; Teske et al., 2002). Usually, hydrothermal and seep habitats generally harbor specialized methane-and sulfur-cycling microbial communities with reduced diversity (Lloyd et al., 2010; Ruff et al., 2015). Recent metagenomic studies of Guaymas surficial sediments are uncovering unexpected microbial diversity, including major phylogenetic lineages of bacteria and archaea (Dombrowski et al., 2017, 2018; Seitz et al., 2019). The environmental conditions that allow for the coexistence of different metabolic types transform the Guaymas Basin surficial sediments into a biodiversity hotspot that will reward further exploration.

## Data Availability Statement

Sequences are available from GenBank under accession numbers MW775652 – MW775666 for 16S rRNA gene amplicons, MW980147 – MW980167 for bacterial amoA gene amplicons, MW911582 – MW911595 for archaeal amoA gene amplicons, and MW980168 – MW980174 for mcrA gene amplicons. Geochemical data are available from the Biological and Chemical Oceanography Data Management Office at the Woods Hole Oceanographic Institution under Project Number 474317 and can be found under Wegener et al., 2021.

## Author Contributions

BE supervised the DGGE experiments, guided undergraduate student assistants TN, SK, KS, and HT through the experimental procedure and together with BH submitted the DGGE band sequences to GenBank. RP collected the sediment cores and measured the thermal gradients during *Alvin* dive 4868. GW performed the porewater geochemical analyses. AT led the expedition, conceptualized the experiments, performed the phylogenetic analyses, and wrote the manuscript. All authors contributed to the article and approved the submitted version.

## Conflict of Interest

The authors declare that the research was conducted in the absence of any commercial or financial relationships that could be construed as a potential conflict of interest.
