# Supplementary material for: Microbial Communities of Hydrothermal Guaymas Basin Surficial Sediment Profiled at 2 Millimeter-Scale Resolution
Source: Front Microbiol. 2021 Jul 16;12:710881. doi: 10.3389/fmicb.2021.710881 (PMC8322767; doi:10.3389/fmicb.2021.710881)
Supplement: Supplementary file 1 [file Data_Sheet_1.pdf]

Supplementary Materials for

Microbial communities of hydrothermal Guaymas Basin surficial sediment profiled at 2 millimeter-scale resolution

B. Engelen, T. Nguyen, B. Heyerhoff, S. Kalenborn, K. Sydow, H. Tabai, R. N. Peterson, G. Wegener, A. Teske

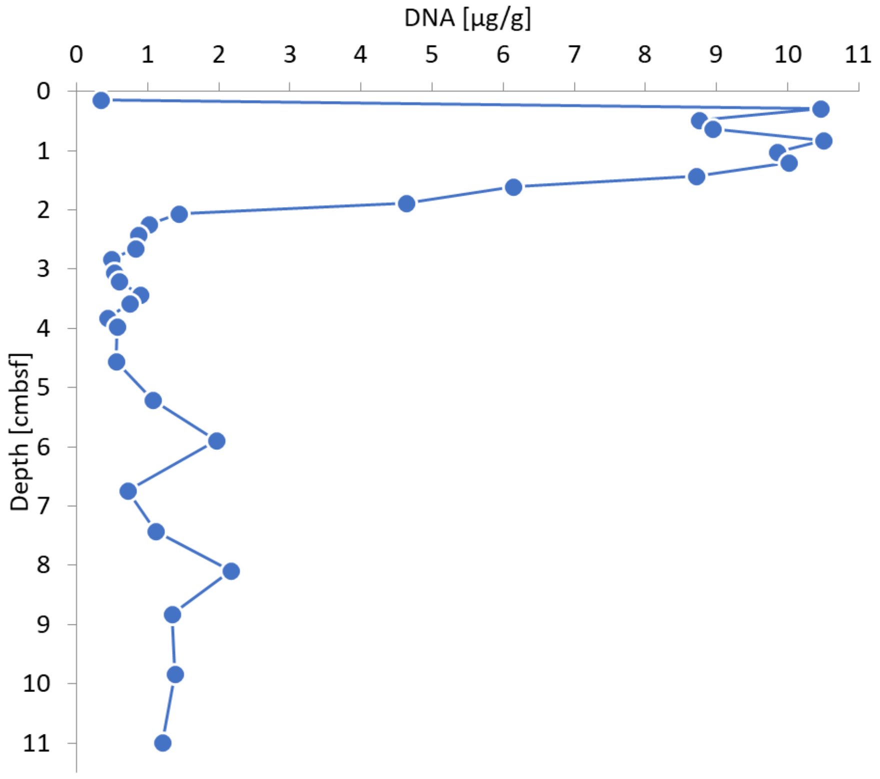

Supplemental Figure 1. DNA concentration of Guaymas core 4868-7. Measured values are based on sediment wet weight.

Supplemental Table 1. In-situ thermal gradient for core 4868-10. Surface temperature is 3°C.

Depth [cmbsf]	Temperature [°C]
10	59.2
20	100.9
30	111.8
40	114.6
50	115.9

Supplementary Table 2. Sequence of 16S rRNA gene DGGE band 1.2, in 3' to 5' orientation, primer sequences excluded.

AGCCTGACGCAGCGACGCCGCGTGGAGGATGAAGGCCTTCGGGTTGTAAACTCCTGTCTGAAGAGAAG
AATTCCCGATTTATCGGGTTGACGGTATCTTCNNAGGAAGCYCCGGCCAACTNCGTGCCANCNNCCGCG
GTAAAACNTAGGGGGcAAACGTTGCTCNGAATTACTGGGCTTAAAGGGRACGCAGGTGGTTAAGCAAG
TCGATAATGAAATCCCGAGGCTCAACTTCGGAATTGTTATCGAAACTGCTTAACTTGAGGATAGTTTAGG
AGAACGGAATTCCCGGTGGARYGGTGAAATGCGTAGATATCGGGAGGAACACCAGAGGCRAAGGCGG
TTCTCTGAACTATTCCTGACMCTGAGGTACGAAAGCTAGGGGAGCAAACGGGATTAGATACCCCGGTA
GTCCTAGCTGTAAACGATGGGCACTANATGTTTCTGTGTTTAKCGGGAGTATCGTAGTTAACGCGTTMA
GTGCCCCGCCTGGGGATTACGGTCGCAAGGMTAAAACTCAAAGGAATTGACGG
